# From laboratory to real-world exposure: correlating cosmetic patch test results with large-scale consumer safety data from e-commerce platforms

**DOI:** 10.3389/fpubh.2026.1791667

**Published:** 2026-04-08

**Authors:** Kai Yang, Qin Zhang, Dangdang Cheng, Feifei Wang

**Affiliations:** 1 Shanghai Jiyan Biomedical Development Co., Ltd., Shanghai, China; 2 Yunnan Botanee Bio-technology Group Co., Ltd., Yunnan, China; 3 Yunnan Yunke Characteristic Plant Extraction Laboratory Co., Ltd., Yunnan, China

**Keywords:** adverse reaction, cosmetics, cosmetovigilance, patch test, sensitive skin

## Abstract

**Background:**

Limited research exists on revealing the correlation between laboratory safety tests and real-world safety feedback from consumers of cosmetics.

**Objective:**

To establish cosmetovigilance by associating patch test results with consumer-reported adverse reaction (CRARs) feedback form e-commerce feedback for cosmetics targeting sensitive skin.

**Methods:**

Patch tests were performed on a cohort of sensitive-skin cosmetics, and CRARs were extracted from e-commerce comments. A generalized linear model was utilized to analyze the association between patch test results and CRARs, considering functional claims and application modes.

**Results:**

Among the patch test results of 64 cosmetics, 42 exhibited negative reactions, 21 showed doubtful reactions, and one cosmetic demonstrated a weak positive reaction. The model established with a warning threshold of two doubtful reactions proved to be optimal. Patch test results were associated with sensitization-related and pain-related CRARs, but not with acnegenic-related CRARs. Brightening and acne treatment cosmetics exhibited higher acnegenic and pain rates, while leave-on cosmetics had a higher sensitization rate.

**Conclusion:**

The data-driven results show that cosmetics with 2 or more doubtful reactions in patch test have a significantly correlation with real-world CRARs, which reminds relevant practitioners to be more cautious about doubtful reactions in patch test.

## 1 Introduction

In the context of the expanding cosmetic market, consumers are not only concerned about the efficacy of products, but also increasingly pay attention to product safety. Studies have indicated that approximately 30 to 50% of consumers experience cosmetic adverse reactions (CARs) to cosmetics (1–4), which manifest as erythema, papules, edema, etc., and are accompanied by symptoms such as itching, burning, and stinging (5, 6). Such adverse reactions may be attributed to certain irritant ingredients (3, 7) and may also be related to the type of product (8). Cosmetic safety has emerged as a global public health concern, with many countries worldwide establishing regulatory systems to monitor cosmetic adverse reactions and mandating companies to report product adverse reactions (9, 10). Companies also establish safety risk assessment systems prior to marketing to reduce risks (11).

Patch test is a well-established method for assessing allergic contact dermatitis. This technique involves applying potential allergens to the skin to evaluate the severity of allergic reactions and detect allergens (12, 13). According to the 2015 edition of the Technical Specifications for Cosmetic Safety issued by the National Medical Products Administration of China, human skin patch test can be utilized to detect the potential for causing skin adverse reactions prior to product marketing. However, current global regulations and studies have not yet clarified the association between patch test results and adverse reactions. In other words, there is a paucity of clear cosmetovigilance for interpreting patch test results. In addition, feedback on adverse reactions after product marketing mainly relies on adverse reaction monitoring systems or the admission records of hospital dermatology departments (14, 15). However, the majority of these records pertain to severe skin adverse reactions, and due to the limitations of data collection methods, the number of reported cases is significantly lower than the actual incidence. In previous studies, consumer comments on adverse reactions of products on social media or e-commerce platforms were often overlooked.

This study retrospectively selected a batch of marketed cosmetics, extracted consumer-reported adverse reaction (CRARs) feedback from e-commerce platforms after marketing, and used existing patch test data of these products. By analyzing the patch test results and CRARs through model analysis, this study retrospectively explore the association between patch test results and CRARs. An referential warning threshold for patch test results was also explored for cosmetics suitable for sensitive skin.

## 2 Material and methods

### 2.1 Sample

A total of 86 marketed cosmetics claiming to be suitable for sensitive skin were included in the study. According to their ingredient lists, all products were free of alcohol, colorants, fragrances, and allergenic ingredients such as methylisothiazolinone and thiomersal, which have been shown in studies to trigger allergies (13, 16, 17). The cosmetics were categorized based on the application modes and functional claims.

### 2.2 Comments on CRARs of marketed products

The extraction and cleansing of consumer comments were conducted based on data provided by a third-party organization. The original comment data was sourced from the annual commentary dataset of the online sales platforms for the year 2023. Following a series of operations, including the removal of duplicates, default comments and extraneous data, consumer comments were obtained. Comments that mentioned the following terms were identified as CRARs comments: ‘irritating', ‘sting', ‘spicy', ‘rash', ‘burning', ‘edema', ‘acne', ‘redness', ‘allergenic', ‘ache,' and ‘itching'. The identification of CRARs was processed by a specifically trained NLP semantic analysis model, which can accurately recognize adverse reaction terms, exclude negations, hypothetical descriptions, and irrelevant expressions. Manual verification was also performed on a random subset of comments to ensure classification accuracy. Correspondence analysis (CA) was performed to analyze and categorize the word frequency of CRARs across all products.

### 2.3 Occluded patch test

The patch tests had been conducted from September 2023 to June 2024, with participants selected according to the inclusion criteria for each batch, with a minimum of 30 participants. Finn Chambers^®^ on Scanpor^®^ 8 mm patch test chambers (Epitest Ltd Oy, Finland) were utilized, providing an area of 50 mm^2^ and a volume of 20 μL.

The test procedure is outlined as follows. The test substance was placed into the chamber of the patch tester, with an amount of approximately 0.020 to 0.025 g for solids or semi-solids, or 0.020 to 0.025 ml for liquids. When the test article was the original cosmetic product, the control well was a blank control (no substance), and when the test article was the diluted cosmetic (rinse-off cosmetics diluted to 1%), the diluent of the cosmetics (distilled water) was used in the control well. The patch tester with the test article was applied to the back of the participant using hypoallergenic tape, and gentle palm pressure was used to ensure even application on the skin for 1 day. Skin reactions were observed 30 min after removal of the patch tester (once the indentation disappeared), and on D2 and D3, with the results being recorded. The patch test and relevant statistical methods followed international recommendations (18), and the criteria for determination are shown in Table 1.

**Table 1:** Scoring system for patch test.

Scoring grades	Symptoms
Negative reaction (–)	/
Doubtful reaction (±)	Faint erythema only
Weak positive reaction (+)	Non-vesicular erythema, infiltration, possibly papules
Strong positive reaction (++)	Vesicular erythema, infiltration, and papules
Extreme positive reaction (+++)	Intense erythema and infiltration, coalescing vesicles, bullous reaction

The above trials were conducted in Shanghai, China in accordance with local legal requirements, and principles of the Good Clinical Practice. This study was approved by an independent ethics committee (approval number: JYE20230908008, JYE20231214012, JYE20240321002, JYE20240530004), which adhered to the International Declaration of Helsinki. All participants have provided written informed consent.

### 2.4 Generalized linear model (GLM)

Cosmetovigilance was established for the patch test results: when the number of reactions for a product exceeded (or was equal to) the warning threshold, the product failed the patch test. The patch test results were processed into binary data (0 for pass, 1 for fail) based on the warning threshold. Three separate GLMs were established, each based on a different warning threshold. The independent variables included the patch test results, the application modes, and the functional claims. The dependent variable was the incidence of post-marketing CRARs. Since the post-marketing CRARs rate was non-count data and did not conform to a normal distribution, a gamma-logarithmic link model was selected. To evaluate the models, we assessed the effects of the independent variables (patch test results, application modes, and functional claims) on the dependent variable (incidence of post-marketing CRARs). Additionally, we compared the Bayesian Information Criterion (BIC) across the models, a smaller BIC value indicates a better model fit.

### 2.5 Statistics

Statistical analysis was conducted using IBM SPSS Statistics 26.0. CA was performed using XLSTAT 2019.2.2. The alpha level for all tests was set at 0.05, with a two-tailed significance.

## 3 Results

### 3.1 Basic information

In this study, a total of 1,803,826 effective consumer comments from e-commerce platforms for 86 products in 2023 were extracted. A total of 22 products with fewer than 1,000 effective comments were excluded in this study, resulting in a final total of 64 products and 1,789,068 effective comments. Among these, 8,793 comments mentioned CRARs, accounting for 0.49%. The 64 products could be categorized into 11 rinse-off cosmetics (RC) and 53 leave-on cosmetics (LC) based on their application modes. They were also categorized into 11 brightening and acne treatment cosmetics (BAC) and 53 other cosmetics (OC) based on their functional claims.

### 3.2 Word frequency analysis of CRARs

**Figure 1:**
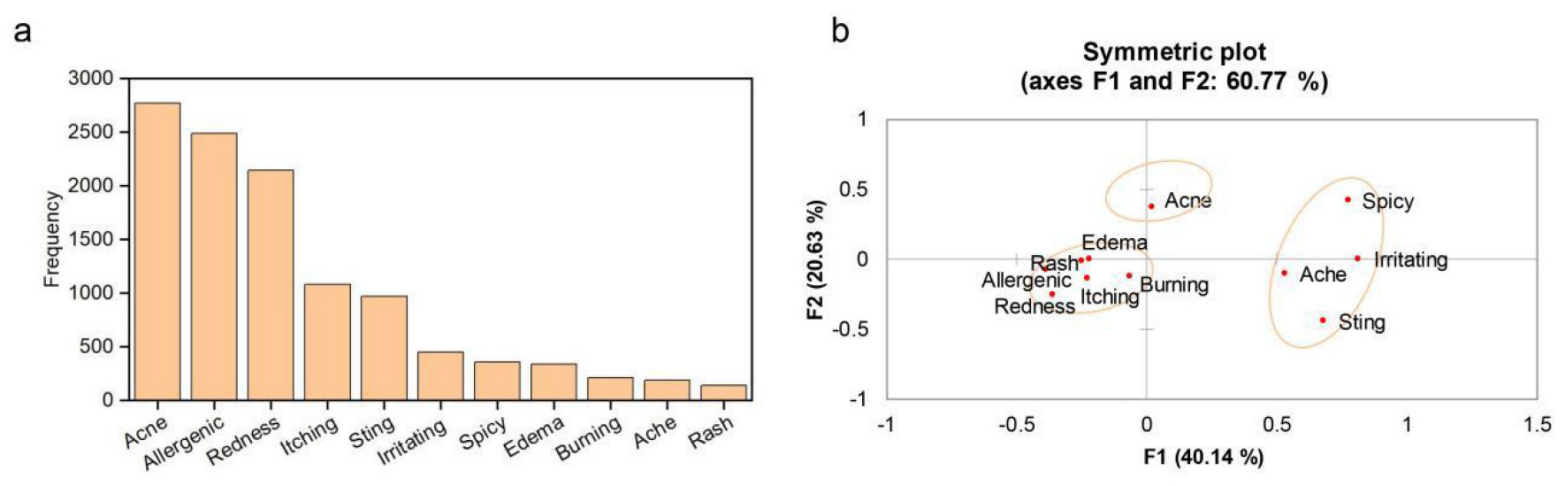
Word frequency bar chart and symmetric correspondence analysis plot of CRARs. **(a)** Frequency distribution of CRARs terms. **(b)** Symmetric plot of correspondence analysis (axes F1 and F2 explain 60.77% of total variance), showing clustering of CRARs symptoms.

The study tallied the occurrence of adverse reaction terms and their frequencies in 8,793 CRARs comments, as detailed in Figure 1a. The three most frequently occurring terms were ‘acne,' ‘allergenic,' and ‘redness,' accounting for 31.53%, 28.32%, and 24.38% of the total CRARs comments, respectively (Figure 1a). Consumers are unable to describe skin symptoms accurately like professionals or medical personnel, necessitating categorization of consumer feedback. A CA was conducted on adverse reaction terms, and Figure 1b revealed potential associations and classification bases among adverse reaction terms. Terms such as ‘allergenic,' ‘itching,' ‘redness,' ‘burning,' ‘rash,' and ‘edema' tended to co-occur and were uniformly classified as sensitization-related symptoms. Terms like ‘irritating,' ‘spicy,' ‘ache,' and ‘sting' primarily involved subjective sensations and were uniformly classified as pain-felated symptoms in this study. ‘Acne' was treated as a separate category. Ultimately, CRARs were categorized into three classes: sensitization-related CRARs, pain-related CRARs and acnegenic-related CRARs. The sensitization rate, pain rate, and acnegenic rate for each product were calculated using the following formulas:


$$\begin{array}{l} R_{S}=\frac{\sum \left(N_{Itching},N_{Rash},N_{Burning},N_{Edema},N_{Redness},N_{Allergenic}\right)}{N_{CC}}\\ R_{P}=\frac{\sum \left(N_{Irritating},N_{Sting},N_{Spicy},N_{Ache}\right)}{N_{CC}}\\ R_{A}=\frac{N_{Acne}}{N_{CC}} \end{array}$$

R_S_: Sensitization rate; R_P_: Pain rate; R_A_: Acnegenic rate; N_CC_: Amount of consumer comments.

### 3.3 Occluded patch test results

In the patch test results of the 64 products, 42 exhibited negative reactions, 21 demonstrated doubtful reactions, and 1 showed a weak positive reaction. The distribution is detailed in Table 2.

**Table 2:** Occluded patch test results of 64 products.

Scoring grades	Number of participants	Number of products	Percentage
Negative reaction (–)	/	42	65.63%
Doubtful reaction (±)			
	1	8	12.50%
	2	7	10.94%
	>2	6	10.94%
Weak positive reaction (+)	/	1	1.47%

### 3.4 GLM analysis

In the process of constructing the GLMs, given that the patch test results of the majority of products in this study were negative or doubtful reactions. Therefore, we chose the number of doubtful reactions as the basis for setting the warning threshold. The three models were established as followed: Model 1: the GLM based on a warning threshold of 1 doubtful reaction; Model 2: the GLM based on a warning threshold of 2 doubtful reactions; Model 3: the GLM based on a warning threshold of 3 doubtful reactions. In addition, multicollinearity among independent variables was assessed via pairwise correlations. All correlation coefficients were below |r| < 0.4, indicating no potential bias in the model results.

For the sensitization rate, patch test results were significant only in Model 2. For the pain rate, significant associations were observed in both Model 1 and Model 2. For the acnegenic rate, no significant association was found in any of the three models. We further compared BIC values across models to identify the optimal model.

**Figure 2:**
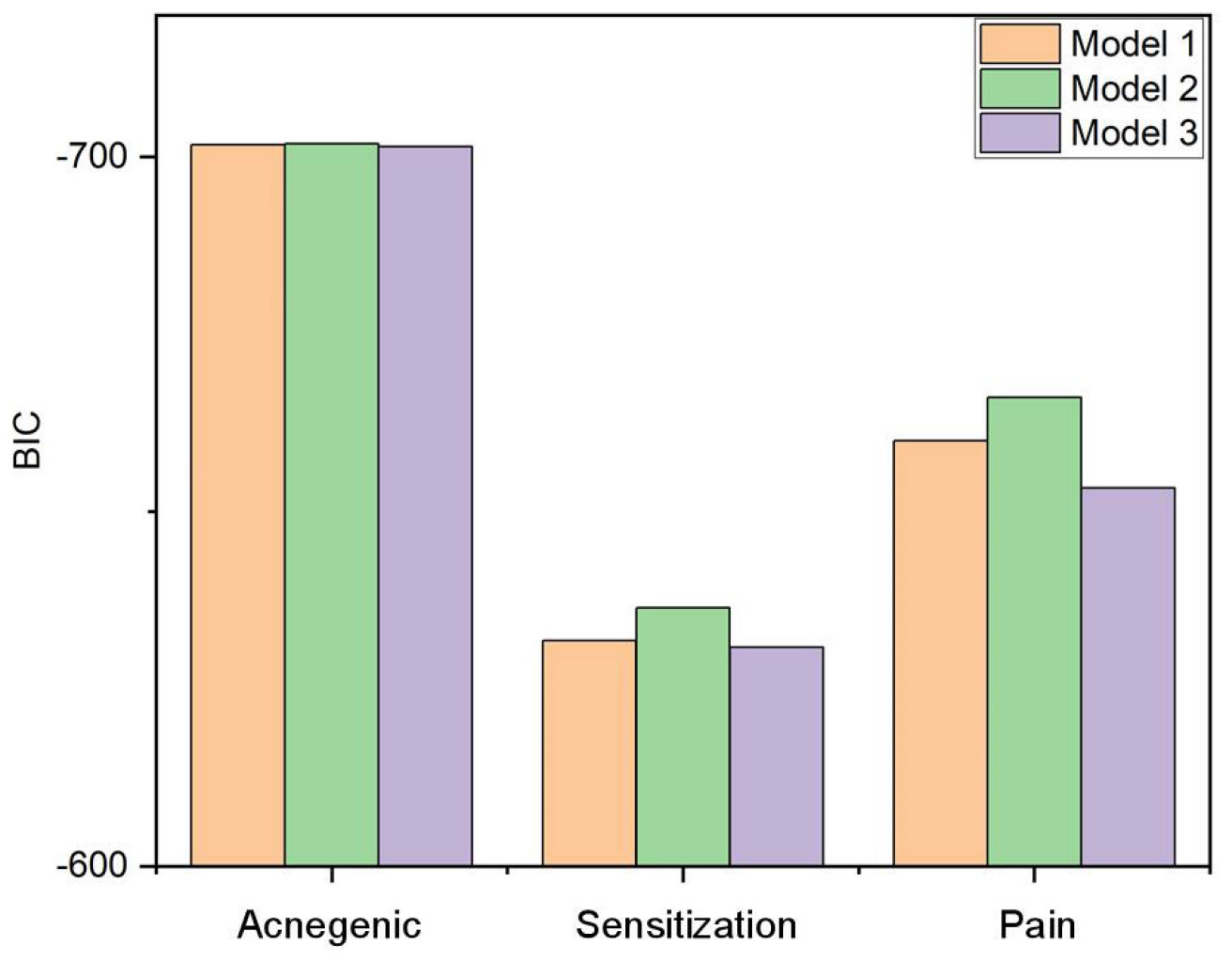
Comparison of Bayesian Information Criterion (BIC) values across three generalized linear models (GLMs) for acnegenic-related, sensitization-related, and pain-related CRARs. Model 1: warning threshold at 1 doubtful reaction; Model 2: warning threshold at 2 doubtful reactions; Model 3: warning threshold at 3 doubtful reactions. Lower BIC values indicate better model fit. Sample size: *n* = 64 products.

When associating with acnegenic reactions, Model 2 exhibited a BIC value of −701.840, which was lower than the BIC values of Model 1 and Model 3 (−701.720 and −701.470, respectively) (Figure 2). For sensitization reactions, Model 2 exhibited a BIC value of −636.439, which was lower than those of Model 1 and Model 3 (−631.800 and −630.870, respectively) (Figure 2). In the case of pain reactions, Model 2 exhibited a BIC value of −666.107, which was lower than the BIC values of Model 1 and Model 3 (−659.958 and −653.299, respectively) (Figure 2). Collectively, Model 2 emerged as the optimal model.

**Figure 3:**
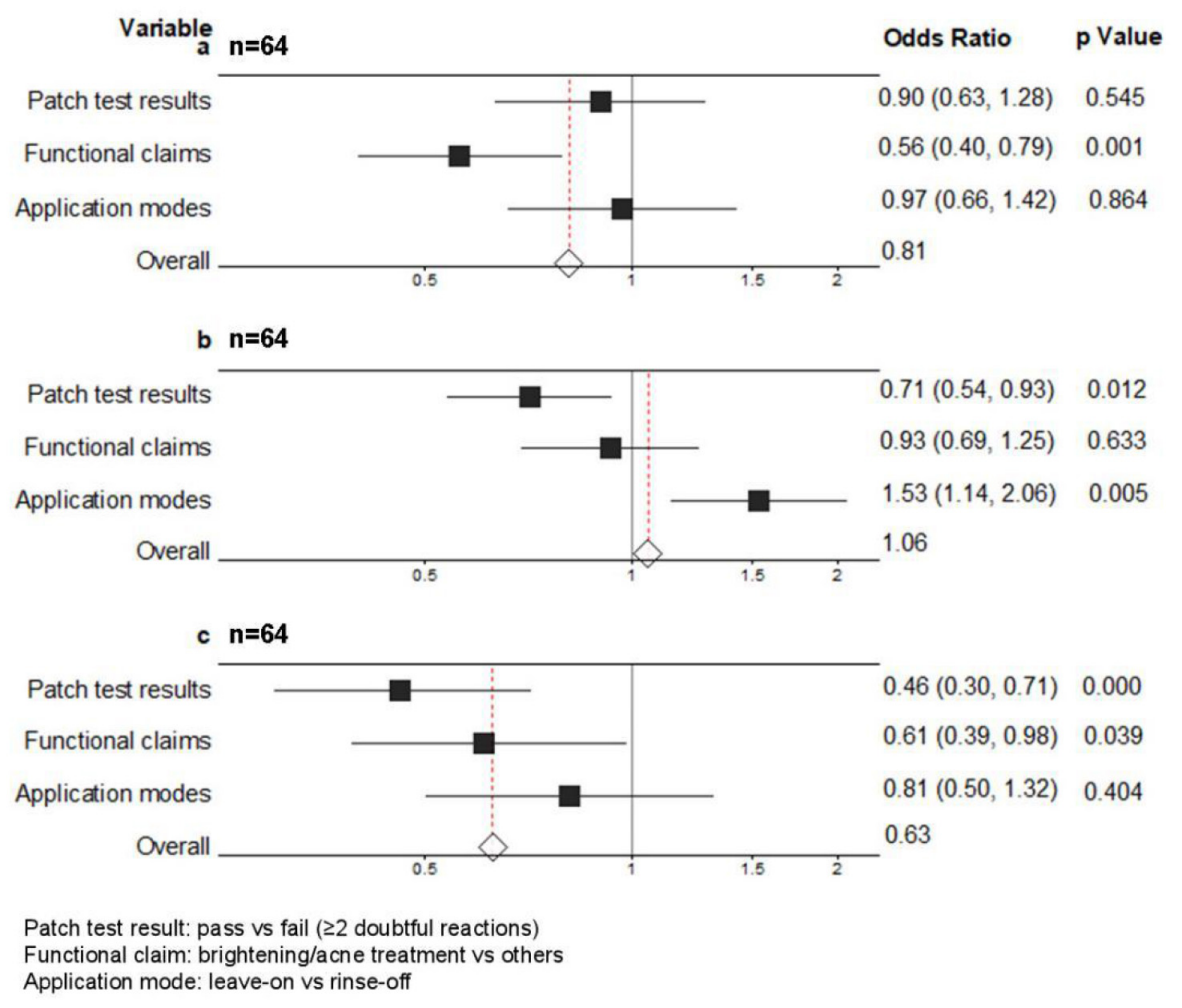
Forest plot of associations between patch test results, functional claims, and application modes and CRARs rates (acnegenic-related, sensitization-related, and pain-related CRARs) based on Model 2 (Gamma generalized linear model with log link, warning threshold: ≥2 doubtful reactions). Odds ratios (OR) and 95% confidence intervals (CI) are presented. **(a)** Acnegenic-related CRARs. **(b)** Sensitization-related CRARs. **(c)** Pain-related CRARs. The middle vertical line is the null line, i.e., OR = 1, indicating that there was no statistically significant association between study factors and outcome. The squares are point estimates of the OR value of the included independent variable, the horizontal line where the square is located represents the 95% confidence interval of the OR value, the red line represents the total combined estimate, and the diamond represents the 95% confidence interval of the combined value.

**Figure 4:**
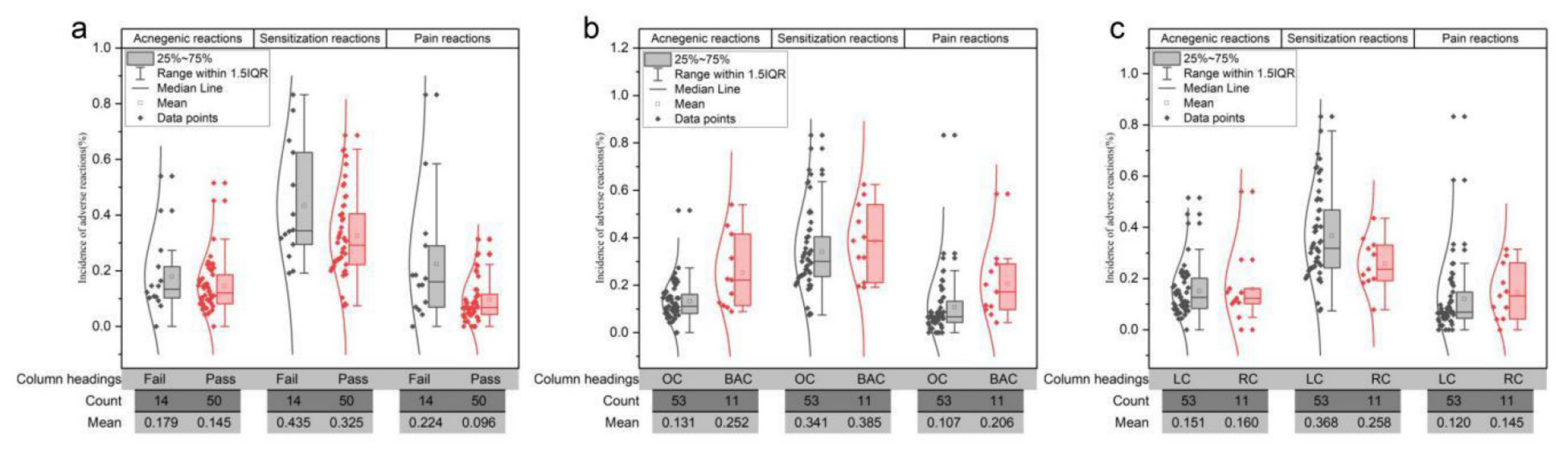
CRARs rates (acnegenic rate, sensitization rate, and pain rate) of products with different patch test results, different functional claims and different application modes under Model 2. **(a)** CRARs rate of products with different patch test results. **(b)** CRARs rate of products with different functional claims. **(c)** CRARs rate of products with different application modes. The curve on the left represents a normal distribution. Fail: When the warning threshold is 2 cases of doubtful reaction, the product fails the patch test result; Pass: When the warning threshold is 2 cases of doubtful reaction, the products that pass the patch test result. BAC, brightening and acne treatment cosmetics; OC, other cosmetics; LC, leave-on cosmetics; RC, rinse-off cosmetics.

As for Model 2, the patch test results were not significantly associated with the acnegenic rate (Figure 3a), but were significantly associated with both the sensitization rate and the pain rate (Figures 3b, c). As shown in Figure 4a, the sensitization rate and pain rate of products that passed the patch test were significantly lower than those that failed, while there was no significant difference in the acnegenic rate. The functional claims had no significant impact on the sensitization rate (Figure 3b), but had a significant impact on both the acnegenic rate and pain rate (Figures 3a, c). As shown in Figure 4b, the acnegenic rate and pain rate of brightening and acne treatment cosmetics were significantly higher than those of other cosmetics, while there was no significant difference in the sensitization rate. The application modes of products had no significant effect on the acnegenic rate and pain rate (Figures 3a, c), but had a significant effect on the sensitization rate (Figure 3b). As shown in Figure 4c, the sensitization rate of leave-on cosmetics was significantly higher than that of rinse-off cosmetics, while there was no significant difference in the acnegenic rate and pain rate.

## 4 Discussion

This retorspective study aimed to establish cosmetovigilance by associating patch test results with CRARs feedback from e-commerce platforms. Previous studies typically presented patch test results as positive rates. However, research has shown that the positive rate of patch tests for marketed cosmetics is less than 5%, which is significantly lower than the proportion of consumers experiencing CRARs when using these products (8, 19). The absence of positive reactions in patch tests does not imply a lower incidence of CRARs. Relying solely on positive rates as a reference for patch test results is no longer sufficient for increasingly stringent safety requirements of consumers, particularly for consumers with sensitive skin who are more prone to allergic symptoms.

In this study, 64 marketed cosmetics claiming to be suitable for sensitive skin were included. Patch tests showed only 1 cosmetic had a weakly positive reaction, likely due to its brightening benefit, which past research has linked to higher rates of positive patch test results (8). The vast majority of the remaining products showed negative patch test results, with a few exhibiting doubtful reactions. Although doubtful reactions in patch tests are typically not used as criteria for determining allergens, many consumers experience mild CRARs, such as faint erythema or other minor symptoms, which align with the symptoms of doubtful reactions observed in patch tests (20). Ronni Wolf (21) contended that doubtful reactions may represent potential safety risks to some extent, and despite ongoing discussions and controversies, they should not be overlooked in research. In recent years, some studies have included doubtful reactions as an important statistical parameter (22). For these reasons, our study considered the number of doubtful reactions of the products as the warning threshold for patch test results.

A total of 8,793 CRARs comments from 64 products on e-commerce platforms in 2023 were retrospectively extracted, accounting for 0.49% of total comments. Compared to adverse reaction monitoring systems that rely on enterprise reporting and collect serious adverse reactions such as skin allergy, photosensitivity, severe skin irritation, and atopic dermatitis, consumer comment data is extensive and easier to obtain. These comments predominantly described mild uncomfort experience, such as redness or skin discomfort, which are more consistent with the doubtful reactions observed in patch tests. However, these comments are based on consumers' actual experiences and subjective judgments, lacking the strict and accurate classification of adverse reactions found in monitoring systems. Therefore, we categorized those CRARs comments into three classes: sensitization-related CRARs, pain-related CRARs, and acnegenic-related CRARs, and established GLMs to associate these with patch test results, functional claims, and application modes.

The results indicated that sensitization and pain-related CRARs significantly increased when products exhibited two or more doubtful reactions in patch tests. In contrast, there was no significant difference in CRARs feedback between patch-test-negative products and those with only one doubtful reaction. This may be attributed to some ingredients defined as low-risk but perform poorly in patch tests, such as ethylhexylglycerin (23). This ingredient is widely used in the tested products, and may cause occasional mild reactions in patch tests, without leading to obvious differences in real-world consumer feedback. Such mild reactions could easily be overlooked without targeted screening. Our study did not conduct a comprehensive screening of product ingredients; therefore, whether the doubtful reactions in patch tests were caused by other hidden low-risk ingredients remains to be further investigated. Additionally, the causes of pain related to cosmetics are complex, including both allergic and irritant pain. Allergic pain is an immune response triggered by an allergen, leading to the release of inflammatory mediators and irritation of nerve endings, while irritant pain results from certain ingredients acting directly on skin nerve receptors (24). For individuals with sensitive skin and compromised skin barriers, both types of pain may coexist, but it is currently unclear which type of pain is more strongly associated with patch test results, necessitating further exploration. Finally, it is not surprising that patch test results did not have a significant impact on the acnegenic rate of products, as consumer comments regarding acne typically refer to acne caused by oil secretion, which is outside the scope of patch test assessments. Although no significant association was found between patch test results and acnegenic rate, acnegenic reactions are common clinical complaints. The lack of significant association may reflect the multifactorial nature of acne, including individual susceptibility, product occlusion, and long-term use patterns, rather than a lack of clinical relevance (25).

These findings are consistent with previous research that emphasizes the importance of patch tests in predicting potential allergic contact dermatitis from cosmetics (12, 13). It is important to clarify that doubtful reactions in patch tests and consumer-reported mild adverse events (mild CRARs) are different biological phenomena with distinct exposure conditions and mechanisms. This study explicitly defines doubtful reactions as exploratory risk signals for cosmetics, not as a direct marker or equivalent of clinical sensitization. However, our study extends these findings by providing quantitative thresholds for the number of doubtful reactions that should be of concern, which is particularly important for product safety assessment and risk management.

This study also revealed that application modes and functional claims significantly impact the incidence of CRARs. Leave-on cosmetics exhibited a higher sensitization rate than rinse-off cosmetics, which is likely attributed to their prolonged contact with the skin. It should be noted that although rinse-off cosmetics were diluted in patch tests, this approach does not accurately reflect real-world consumer usage scenarios. Therefore, while some previous studies have reported that rinse-off cosmetics have a higher positive rate in patch tests compared to leave-on cosmetics, the number of CRARs reports associated with rinse-off cosmetics was far fewer than those for other categories (26). This finding highlights the necessity for more rigorous safety assessments for products intended for prolonged use on the skin. Additionally, the results indicate that brightening and acne treatment cosmetics had higher rates of acnegenic and pain-related CRARs. This observation may be attributed to the presence of irritating ingredients in the formulations of these products. Further investigation into the ingredients of cosmetics is warranted to identify potential allergens and guide the development of safer formulations.

Overall, for cosmetics designed for sensitive skin, this study found that the risk of sensitization and pain-related CRARs feedback significantly increases when two or more doubtful reactions were observed in patch tests. Specifically, brightening and acne treatment cosmetics were found to exhibited a higher risk of acnegenic and pain-related CRARs, whereas leave-on cosmetics exhibited a greater propensity for sensitization-related CRARs.

A notable strength of our study is the large sample size of consumer feedback and the comprehensive analysis of patch test results, which enabled the establishment of robust associations between CRARs and patch tests results. Furthermore, the establishment of cosmetovigilance using GLMs adds a quantitative dimension to the interpretation of patch test results, representing a novel approach in the field of cosmetic safety.

However, this study has several limitations that should be acknowledged. At the e-commerce data level, this study relied on consumer feedback from e-commerce platforms for CRARs data, which may introduce bias given that not all consumers report CRARs; while a sufficiently large sample size helps mitigate some of this bias, the findings may be less applicable to products with fewer consumer comments. At the experimental control level, this study only recorded the presence or absence of CRARs, without grading their severity—a limitation that will be addressed in future research. And potential confounding factors, such as formulation complexity, ingredient concentrations, brand quality, consumer skin types, daily usage patterns, and individual differences in skin sensitivity, were not fully controlled. At the application and interpretation level, as this is a product-level ecological study, the possibility of ecological fallacy cannot be ruled out; the results thus reflect product-level safety signals rather than risks at the individual level. Additionally, the warning threshold identified here is exploratory and data-driven, based on the current sample, and should not be broadly generalized to other cosmetic types or populations without further validation, as this could lead to overfitting. Despite these limitations, this study offers a practical framework for integrating patch test results with consumer feedback to support cosmetic safety surveillance. Extrapolating these product-level safety signals to individual risk prediction requires caution, and further validation across diverse consumer populations is needed to improve generalizability.

In conclusion, this study provides valuable insights into the relationship between patch test results and CRARs for cosmetics. Specifically, a significant incidence of sensitization-related and pain-related CRARs was observed in products that exhibited two or more doubtful reactions in patch tests. This finding indicates that relevant practitioners should pay greater attention to doubtful reactions observed in patch tests, thereby facilitating the early identification of potential safety risks of cosmetics. This approach contributes to reducing the incidence of CRARs and improving the safety of cosmetics for consumers with sensitive skin.

## Data Availability

The data analyzed in this study is subject to the following licenses/restrictions: Data are available on request to the authors. Requests to access these datasets should be directed to yangkai@botanee.com.
